# Intratumoral susceptibility signals in adult-type diffuse gliomas: associations with molecular profile, tumor grade, and overall survival

**DOI:** 10.3389/fmed.2026.1905588

**Published:** 2026-10-14

**Authors:** Paola Feraco, Diego Giuliani, Giuseppe Espa, Cesare Gagliardo, Dylan Henssen, Frederick J. A. Meijer, Valentina Greco, Giovanni Bertalot, Orazio Caffo

**Affiliations:** 1 Dipartimento di Medicina e Scienze della Salute, University of Trento, Trento, Italy; 2 Neuroradiology Unit, Santa Chiara Hospital, ASUIT, Trento, Italy; 3 Department of Economy and Management, University of Trento, Trento, Italy; 4 Radiomics Laboratory, Department of Economy and Management, University of Trento, Trento, Italy; 5 Department of Biomedicine, Neurosciences and Advanced Diagnostics (BIND), University of Palermo, Palermo, Italy; 6 Department of Medical Imaging, Radboud University Medical Center, Nijmegen, Netherlands; 7 Department of Nuclear Medicine, University Hospital Leipzig, Leipzig, Germany; 8 Medical Oncology Department, Santa Chiara Hospital, ASUIT, Trento, Italy; 9 Surgical Pathology, Santa Chiara Hospital, ASUIT, Trento, Italy

**Keywords:** glioma, intratumoral susceptibility signals (ITSS), isocitrate dehydrogenase, MRI, radiogenomic

## Abstract

**Purpose:**

Intratumoral Susceptibility Signals (ITSS) are low-signal-intensity structures observed on susceptibility-weighted imaging (SWI) which may reflect intratumoral microhemorrhages, calcifications, and neovascularization. Previous studies have demonstrated associations between ITSS, glioma grade, and molecular characteristics; however, their potential role in outcome prediction remains incompletely defined. This study investigated the diagnostic and and potential prognostic role of ITSS in adult diffuse gliomas.

**Methods:**

We retrospectively analyzed 128 adult patients with histologically confirmed diffuse gliomas who underwent preoperative MRI including SWI. ITSS were graded from 0 to 3 according to the number of susceptibility signals. Associations between ITSS grade and CNS WHO tumor grade, IDH mutation status, MGMT promoter methylation and Ki-67 labeling index (LI) were evaluated. Overall survival (OS) was assessed using Kaplan–Meier analysis and Cox proportional hazards models.

**Results:**

ITSS grade was significantly associated with WHO glioma grade (*p* < 0.001), with lower-grade tumors predominantly exhibiting ITSS grades 0–1 and higher-grade gliomas showing grades 2–3. IDH-mutant gliomas displayed significantly lower ITSS grades than IDH-wildtype tumors (*p* < 0.001). ITSS grade was positively correlated with Ki-67 labeling index (Spearman's *ρ* = 0.423, *p* < 0.001). In the survival cohort (*n* = 87) higher ITSS burden was significantly associated with shorter OS (ITSS 2–3 vs. 0–1: HR = 3.19, 95% CI 1.65–6.19; *p* < 0.001). However, this association was no longer significant after adjustment for established prognostic factors (HR = 1.40, 95% CI 0.69–2.86; *p* = 0.350), with *IDH* status emerging as the strongest independent association with shorter OS.

**Conclusion:**

ITSS grading on SWI is associated with CNS WHO grade, molecular profile, proliferative activity, and OS in adult-type diffuse gliomas. Although ITSS was not an independent predictor of survival after adjustment for established prognostic factors, it may serve as a readily available, non-invasive imaging correlate of tumor aggressiveness, providing complementary information for preoperative tumor characterization while definitive molecular data are pending.

## 1 Introduction

Gliomas are the most common primary malignant brain tumors in adults, comprising approximately 80% of malignant CNS tumors. Their incidence is estimated at 6 per 100,000 people annually, with glioblastomas accounting for the majority of cases (1). Despite advances in therapy, prognosis remains poor, particularly in high-grade variants. The therapeutic management of gliomas, defined within a multidisciplinary framework, is contingent upon multiple variables, including tumor histotype, anatomical location, grade of malignancy, and patient-specific factors such as age and performance status. A precise and comprehensive characterization of central nervous system (CNS) neoplasms is essential to inform and optimize treatment strategies. In the context of precision oncology, molecular and genetic profiling has assumed a central role, offering critical insights into tumor pathobiology. The 2016 (4th edition) and 2021 (5th edition) updates of the World Health Organization (WHO) classification of CNS tumors have introduced a paradigm shift by integrating molecular diagnostics into the histopathological classification of gliomas. This integrated approach, which transcends conventional morphology-based taxonomy, enables a more refined and biologically meaningful stratification of glioma entities (2–4). According to the 2021 WHO Classification, definitive diagnosis of gliomas relies on a combination of histopathologic features and molecular markers, typically assessed via immunohistochemistry, polymerase chain reaction (PCR), and/or fluorescence *in situ* hybridization (FISH). Current classification of diffuse gliomas primarily hinges on the identification of two key molecular alterations: mutations in isocitrate dehydrogenase (*IDH*) genes and co-deletion of chromosomal arms 1p and 19q (2–4). Additional molecular biomarkers with established diagnostic and prognostic significance include mutations in the promoter region of the telomerase reverse transcriptase (TERT) gene, O6-methylguanine-DNA methyltransferase (MGMT) promoter methylation status, Ki-67 proliferation index, and inactivation of the ATRX (alpha-thalassemia/mental retardation syndrome X-linked) gene (5–8). Definitive diagnosis, grading, and subtype delineation of gliomas are traditionally achieved through histopathological evaluation of tissue specimens obtained via stereotactic biopsy or surgical resection (4). Nevertheless, neuroradiological assessment, predominantly through magnetic resonance imaging (MRI) with conventional and advanced sequences, plays a pivotal role in the preoperative characterization of gliomas. Neuroradiological features may offer indirect but valuable clues regarding the underlying molecular profile and histological grade of the tumor (9, 10).

Over the past decades, substantial research efforts have been directed toward enhancing glioma characterization through advanced neuroimaging modalities, such as MR spectroscopy, diffusion, and perfusion imaging (11, 12). More recently, Susceptibility-Weighted Imaging (SWI) has garnered increasing attention and is progressively being incorporated into standard neuro-oncological imaging protocols, owing to its heightened sensitivity in detecting paramagnetic substances such as deoxyhemoglobin, hemosiderin, and calcium (13). Within SWI sequences, Intratumoral Susceptibility Signals (ITSSs) are defined as low-signal-intensity linear or punctate foci, either isolated or conglomerated, reflecting various pathological substrates (14). Park et al. were the first to propose a semi-quantitative grading system (from grade 0 to grade 3) for ITSSs based on their number in the most representative axial slice (15). Histopathologically, ITSSs correlate with microhemorrhages, microcalcifications, necrotic foci (manifesting as dot-like hypointensities), and aberrant neovascularization (appearing as fine linear hypointensities) (15). Notably, high-grade gliomas (HGGs) exhibit a higher density and complexity of ITSSs compared to low-grade gliomas (LGGs), a finding attributable to their angiogenic dysregulation, increased microvascular fragility, and propensity for necrosis and hemorrhage (16). Moreover, despite the recognized association between ITSS and glioma aggressiveness, the clinical implications of these findings remain incompletely defined. In particular, it is unclear whether ITSS can contribute additional information for patient stratification when integrated with established molecular and histopathological markers. Accordingly, the aim of this study was to assess the diagnostic value of ITSS for glioma grading and molecular characterization and to explore its association with overall survival (OS) in adult diffuse gliomas.

## 2 Material and methods

### 2.1 Patient cohort

This retrospective study was approved by the local Ethics Committee. A consecutive cohort of 139 adult patients who underwent surgical resection between 2014 and 2020 was initially screened for inclusion. The study population was restricted to adult patients (age ≥18 years), as our center does not perform neurosurgical procedures for pediatric brain tumors. Inclusion criteria were: (1) age ≥18 years; (2) histopathologically confirmed diagnosis of diffuse glioma according to the 2021 WHO classification; (3) availability of preoperative conventional MRI (cMRI), including susceptibility-weighted imaging (SWI) sequences; and (4) availability of progression-free survival (PFS) and overall survival (OS) data. Exclusion criteria were: (1) lack of histopathological data and (2) incomplete MRI examinations.

### 2.2 MRI acquisition and image analysis

MRI scans were acquired as part of routine clinical assessment using a 1.5 Tesla MR system (GE Optima MR450w 1.5 T, Waukesha, WI, USA) equipped with an 8-channel phased-array head coil. The standard cMRI protocol included: axial T1-weighted fast spin-echo (FSE) sequence (TR = 630 ms; TE = 4.2 ms), axial T2-weighted fast relaxation fast spin echo—periodically rotated overlapping parallel lines with enhanced reconstruction (FRFSE-PROPELLER) sequence (TR = 5300 ms; TE = 96 ms; NEX = 3.3), axial T2-weighted fluid-attenuated inversion recovery (FLAIR) sequence (TR = 11,000 ms; TE = 92 ms; TI = 2,775 ms). For all sequences: slice thickness = 4 mm, slice overla*p* = 1 mm, and field of view (FOV) = 240 × 240 mm.

A 3D susceptibility-sensitive imaging (Susceptibility Weighted ANgiography, i.e., SWAN protocol: TR, minimum value of ≈ 80 milliseconds; TE, 50 milliseconds; flip angle, 25°; echo-train length, 10; slice thickness, 3 mm; slice overlap, 1.5 mm; frequency phase, 320/256; reconstructed voxel size, 0.47 × 0.47 × 3 mm; acquisition time, 4 min 40 s) was acquired to evaluate ITSS providing both magnitude and phase information.

ITSS grading was assessed by two European board-certified neuroradiologists with 25 and 15 years of experience in neuroradiology) who were blinded to the histopathological results and molecular data. ITSSs were defined as linear or punctate low-signal intensity foci, either clustered or scattered within the tumor, clearly discernible on SWI compared to conventional sequences. Based on morphological characteristics, ITSSs were categorized into three types: (a) clustered dot-like structures; (b) fine linear structures; (c) mixed pattern (dot-like and linear structures). ITSS grading was performed according to the method described by Park et al. (15), evaluating the cross-sectional image with the greatest number of signals: Grade 0: no ITSSs, Grade 1: 1–5 dot-like or linear ITSSs, Grade 2: 6–10 dot-like or linear ITSSs and Grade 3: ≥11 dot-like or linear ITSSs.

During image assessment, phase information was available to the readers and was used, when necessary, to differentiate diamagnetic susceptibility sources, such as calcification, from paramagnetic sources, including blood products and iron, thereby minimizing the potential contribution of calcification to ITSS grading. CT scans were reviewed, particularly in cases of suspected oligodendroglioma. Each reader assigned the ITSS grade independently. Discrepant cases were subsequently reviewed jointly and resolved by consensus to determine the final ITSS grade used for subsequent analyses.

### 2.3 Pathological and immunohistochemical analysis

Tumor classification and grading were performed in accordance with the 2021 WHO criteria based on the histopathological and molecular information available for each case (2). *IDH* mutational status was assessed via immunohistochemical detection of the R132H variant of *IDH*1, the most prevalent mutation in gliomas. IHCs were performed using Bond III IHC autostainer (Leica biosystems) with the following antibody: R132H variant of *IDH*1s (clone H09) (Dianoca, Germany). The antigen retrieval was performed with BOND™ Epitope Retrieval ER2 Solution (Leica). Antibody was diluted with Bond Primary Antibody Diluent AR9352 Leica and BOND IHC Polymer Detection Kit (DS9800) was used to stain antibody with DAB Chromogen. For patients under 55 years of age presenting with an IDH1-R132H negative IHC result, but whose histological features strongly suggested an *IDH* mutation, Sanger sequencing of *IDH1* and *IDH2* (exon 4) was performed to screen for non-canonical or rare variants (17).

In *IDH*1-mutant cases, to exclude the presence of 1p/19q codeletion, and thereby a diagnosis of oligodendroglioma, fluorescence *in situ* hybridization (FISH) was performed. The specific methodologies for molecular testing have been described in previous literature (18, 19). Given the retrospective nature of the study and the long inclusion period, the complete panel of molecular alterations currently used for integrated WHO CNS5 classification was not uniformly available across the entire cohort. IDH-wildtype diffuse astrocytic tumors showing histological features of glioblastoma, namely microvascular proliferation and/or necrosis, were classified as glioblastoma, IDH-wildtype, CNS WHO grade 4. In tumors lacking these histological features, molecular parameters were considered when available; the presence of TERT promoter mutation, EGFR amplification, or combined whole-chromosome 7 gain/whole-chromosome 10 loss (+7/−10) supported classification as glioblastoma, IDH-wildtype, CNS WHO grade 4. Tumors reported as grade 2 or 3 were retained according to the available integrated histopathological and molecular diagnosis (2).

The Ki-67 proliferation index was assessed immunohistochemically using clone MIB-1 (Dako, Agilent Technologies). Quantification was performed by calculating the average number of positive cells found in five different high power fields (approximately 500 cells).

MGMT promoter methylation status was assessed on formalin-fixed paraffin-embedded (FFPE) tumor samples using the EasyPGX® Ready MGMT kit (Diatech Pharmacogenetics, Jesi, Italy). Genomic DNA was extracted from FFPE tissue and analyzed by real-time PCR followed by methylation curve analysis. The assay qualitatively evaluates the methylation status of 12 CpG sites located within the MGMT promoter region (hg19 chr10:131,265,494–131,265,555). According to the manufacturer's recommendations, samples showing a methylation level of approximately ≥10% were classified as methylated, whereas those below this threshold were considered unmethylated.

### 2.4 Statistical analyses

Categorical variables (ITSS grade, IDH mutation status, MGMT promoter methylation status, and CNS WHO grade) were analyzed using contingency tables, with associations assessed using the chi-squared (*χ*^2^) test of independence or Fisher's exact test, as appropriate. Effect sizes for categorical associations were quantified using Cramér's V. The association between ITSS grade and contrast enhancement, considered as a binary variable (present/absent), was assessed using the same approach. This analysis was also performed separately within molecular glioma subgroups.

Continuous variables were tested for normality using the Shapiro–Wilk test and are reported as mean ± standard deviation or median and interquartile range (IQR), as appropriate. Given the ordinal nature of the grading scale, inter-observer agreement was assessed using weighted Cohen's *κ*. The association between ITSS and Ki-67 labeling index was assessed using Spearman's rank correlation coefficient (*ρ*). Differences in Ki-67 distribution across ITSS grades were evaluated using the Kruskal–Wallis test.

OS was analyzed using Kaplan–Meier curves, with differences between groups assessed using the log-rank test. ITSS was evaluated both according to the four individual grades (0–3) and after dichotomization into low (grades 0–1) and high (grades 2–3) ITSS burden. Survival analyses were also performed within subgroups defined by IDH mutation status and WHO grade. Cox proportional hazards regression was used to estimate hazard ratios (HRs) and 95% confidence intervals (CIs). ITSS was evaluated in univariate analyses both as an ordinal variable and as a dichotomized variable (grades 2–3 vs. 0–1). Multivariable Cox models were constructed to evaluate the association of ITSS with OS after adjustment for age, sex, and either IDH mutation status or WHO grade. Statistical significance was defined as *p* ≤ 0.05.

## 3 Results

### 3.1 Patient cohort composition

Demographic characteristics of the molecular subgroups are shown in the Table 1. Of the 139 patients initially screened, 11 were excluded due to incomplete immunohistochemical or pre-treatment MRI data, resulting in a final study cohort of 128 adult patients. The age of the patients ranged from 22 to 78 years.

**Table 1:** Demographic characteristics of the molecular subgroups.

Characteristic	IDH-wt	IDH-mut astrocytoma	IDH-mut, 1p/19q-codeleted
*N* (total)	63	46	19
Male, *n* (%)	38 (60.3%)	27 (58.7%)	11 (57.9%)
Female, *n* (%)	25 (39.7%)	19 (41.3%)	8 (42.1%)
Mean age, years	61.4	42.6	44.9
Age SD (±)	10.6	14.1	13.4
Age range, years	38–78	22–74	26–74

Based on genetic and biomolecular profiling, 63 tumors exhibited *IDH*-wildtype (*IDH*-wt) status while 65 tumors harbored *IDH* mutations (*IDH*-mut). Among the 63 IDH-wildtype gliomas, 50 were classified as glioblastoma, IDH-wildtype, CNS WHO grade 4. Of these, 42 tumors showed histological features of grade 4 disease, whereas 8 histologically lower-grade diffuse astrocytic tumors were classified as CNS WHO grade 4 based on the presence of WHO 2021-defining molecular alterations (TERT promoter mutation, EGFR amplification, and/or combined chromosome 7 gain/chromosome 10 loss). The remaining 13 IDH-wildtype tumors (6 grade 2 and 7 grade 3) retained their historical grade because the molecular information required for retrospective CNS WHO 2021 reclassification was incomplete. Among the *IDH*-mutant gliomas, 1p/19q co-deletion was identified in 19 cases. According to the 2021 WHO classification, the final cohort was distributed as follows: 46 *IDH*-mutant astrocytomas, 19 oligodendrogliomas (*IDH*-mutant with 1p/19q co-deletion) and 63 *IDH*-wildtype.

### 3.2 Intratumoral susceptibility signals (ITSS) and molecular markers

Inter-rater agreement for ITSS grading was high (weighted Cohen's *κ* = 0.85), and discrepancies were resolved by consensus. ITSS grade differed significantly among the three molecular glioma subtypes (*IDH*-wildtype, *IDH*-mutant astrocytoma, and oligodendroglioma, *IDH*-mutant and 1p/19q-codeleted; *p* < 0.001; Figure 1, Table 2). No statistically significant association was observed between ITSS grade and MGMT promoter methylation status (*p* = 0.065).

**Figure 1:**
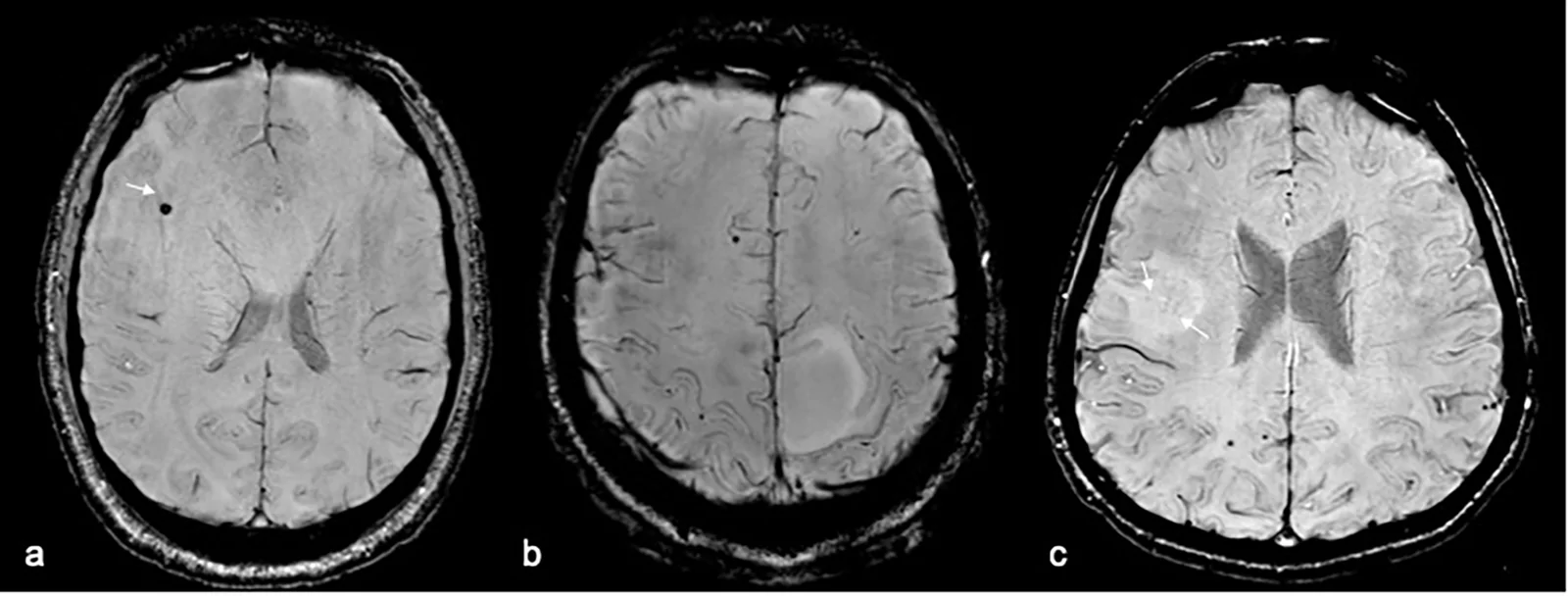
ITSS grading on SWI sequences of the three molecular glioma subtypes. **(a)** ITSS=1, grade 2 *IDH*-mut with 1p/19q co-deletion; **(b)** ITSS =0, grade 2 *IDH*-mut; **(c)** ITSS=2, grade 2 *IDH*-wt. Low-signal-intensity linear or punctate foci are detected in **(a,c)** (white arrows). A statistically significant difference in ITSS distribution was observed between the molecular classes (*p* < 0.001), supporting the association between ITSS and glioma molecular status, even within identical histological grades.

**Table 2:** Distribution of ITSS grades according to IDH mutation status in 128 gliomas.

	ITSS 0	ITSS 1	ITSS 2	ITSS 3	Total
a) IDH status					
Mutant (*n* = 46)	30 (65.2%)	7 (15.2%)	3 (6.5%)	6 (13.0%)	46
Mutant/1p19q codeleted (*n* = 19)	4 (21.1%)	10 (52.6%)	3 (15.8%)	2 (10.5%)	19
Wild-type (*n* = 63)	8 (12.7%)	19 (30.2%)	14 (22.2%)	22 (34.9%)	63
Total	42 (32.8%)	36 (28.1%)	20 (15.6%)	30 (23.4%)	128
b) MGMT status					
MGMT-met	26 (36.1%)	22 (30.6%)	12 (16.7%)	12 (16.7%)	72
MGMT-wt	11 (22.9%)	11 (22.9%)	8 (16.7%)	18 (37.5%)	48
Total	37 (30.8%)	33 (27.5%)	20 (16.7%)	30 (25%)	120

(a) ITSS can discriminate between IDH mutational group (*p* < 0.001); (b) only a trend-level correlation was observed between ITSS grade and MGMT promoter methylation status (*p* = 0.065), appearing more pronounced at the extremes of the ITSS grading scale.

### 3.3 Intratumoral susceptibility signals (ITSS) and contrast enhancement

Contrast-enhancement data were available in 127 of 128 patients. A significant association was observed between ITSS grade and the presence of contrast enhancement (*χ*^2^ = 63.38, *p* < 0.001; Cramér's V = 0.71). The proportion of enhancing tumors increased from 21.4% (9/42) for ITSS grade 0 to 85.7% (30/35), 85.0% (17/20), and 100% (30/30) for ITSS grades 1, 2, and 3, respectively. Nevertheless, ITSS were also identified in eight non-enhancing tumors, including three tumors with ITSS grade 2. When analyzed according to molecular subtype, the association between ITSS grade and contrast enhancement remained significant in both IDH-wildtype gliomas (*p* < 0.001) and IDH-mutant astrocytomas (*p* < 0.001), whereas no significant association was observed in IDH-mutant, 1p/19q-codeleted oligodendrogliomas. Of note, the present analysis assessed the association between the presence of enhancement and ITSS burden at the lesion level and did not imply spatial co-localization of the two imaging features.

### 3.4 ITSS and grade

A robust association was found between ITSS grade and WHO tumor grade (*p* < 0.001, see Table 3). Across the overall cohort, WHO grade 2 gliomas predominantly exhibited ITSS grades 0 (∼56%) and 1 (∼27%). A similar pattern was observed in WHO grade 3 gliomas, though with a slight shift toward higher ITSS grades. In contrast, WHO grade 4 gliomas showed a marked enrichment of ITSS grade 3 (40%), with ITSS 0 observed in fewer than 4% of cases. When stratified by histological subtype, this association remained statistically significant in astrocytomas (*p* = 0.027). Specifically, 85% of WHO grade 2 astrocytomas and ∼57% of grade 3 displayed ITSS 0 (Figure 2). In grade 4 astrocytomas, although the sample size was limited, the ITSS distribution was more heterogeneous, with 40% exhibiting ITSS 3.

**Table 3:** Correlation between ITSS grades and WHO grade in CNS gliomas (*n* = 128).

	ITSS 0	ITSS 1	ITSS 2	ITSS 3	Total
a) General population Grade *(p* *<* *0.001)*					
2	19 (55.9%)	9 (26.5%)	3 (8.8%)	3 (8.8%)	34 (100%)
3	21 (53.8%)	10 (25.6%)	3 (7.7%)	5 (12.8%)	39 (100%)
4	2 (3.6%)	17 (30.9%)	14 (25.5%)	22 (40.0%)	55 (100%)
Total	42 (32.8%)	36 (28.1%)	20 (15.6%)	30 (23.4%)	128 (100%)
b) IDH-mut Grade *(p* *=* *0.084)*					
2	17 (85.0%)	2 (10.0%)	1 (5.0%)	0 (0.0%)	20 (100%)
3	12 (57.1%)	4 (19.0%)	1 (4.8%)	4 (19.0%)	21 (100%)
4	1 (20.0%)	1 (20.0%)	1 (20.0%)	2 (40.0%)	5 (100%)
Total	30 (65.2%)	7 (15.2%)	3 (6.5%)	6 (13.0%)	46 (100%)
c) IDH-mut/1p-19q codeleted Grade					
2	0 (0.0%)	5 (62.5%)	1 (12.5%)	2 (25.0%)	8 (100%)
3	4 (36.4%)	5 (45.5%)	2 (18.2%)	0 (0.0%)	11 (100%)
Total	4 (21.1%)	10 (52.6%)	3 (15.8%)	2 (10.5%)	19 (100%)

(a) A robust association was found between ITSS grade and WHO tumor grade (*p* < 0.001); (b) When stratified by histological subtype, this association remained statistically significant in astrocytomas (*p* = 0.027); (c) while in oligodendrogliomas, the correlation between ITSS and WHO grade did not reach statistical significance.

**Figure 2:**
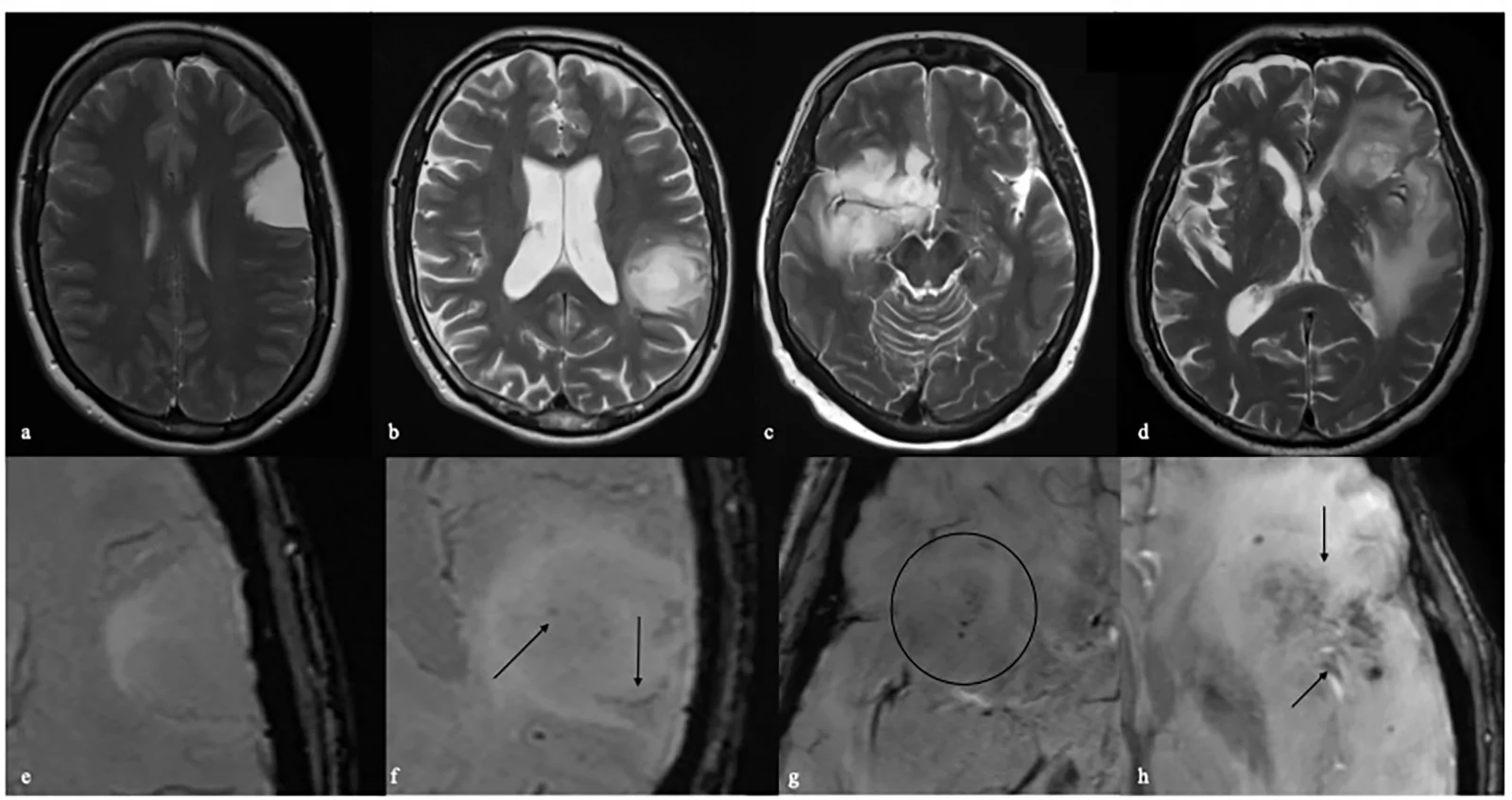
T2w **(a–d)** and susceptibility-weighted imaging (SWI, e-h) of four IDH-mutant gliomas with increasing wHO grades. **(a,e)** WHO grade 2 astrocytoma showing no intratumoral susceptibility signals (ITSS grade 0); **(b,f)** WHO grade 3 astrocytoma with a few punctate hypointense foci (ITSS grade 1, black arrows); **(c,g)** WHO grade 3 astrocytoma with more than six punctate foci (ITSS grade 2, black circle); **(d,h)** WHO grade 4 astrocytoma exhibiting multiple linear and punctate hypointense foci throughout the lesion (ITSS grade 3).

In oligodendrogliomas, the correlation between ITSS and WHO grade did not reach statistical significance. Nevertheless, WHO grade 2 oligodendrogliomas showed no cases with ITSS 0, with a distribution ranging from grades 1 to 3, and a predominance at ITSS 1 (∼63%). Conversely, grade 3 oligodendrogliomas demonstrated ITSS grades ranging from 0 to 2, with no cases exhibiting ITSS 3 and a peak at ITSS 1 (∼46%).

Ki-67 labeling index increased with increasing ITSS burden, with median values of approximately 5%, 15%, 20%, and 20% for ITSS grades 0, 1, 2, and 3, respectively. A significant positive correlation was observed between ITSS grade and Ki-67 labeling index (Spearman's *ρ* = 0.423, *p* < 0.001). Consistently, Ki-67 distributions differed significantly across ITSS grades (Kruskal–Wallis H = 18.97, *p* < 0.001), supporting an association between increasing ITSS burden and higher proliferative activity (Figure 3).

**Figure 3:**
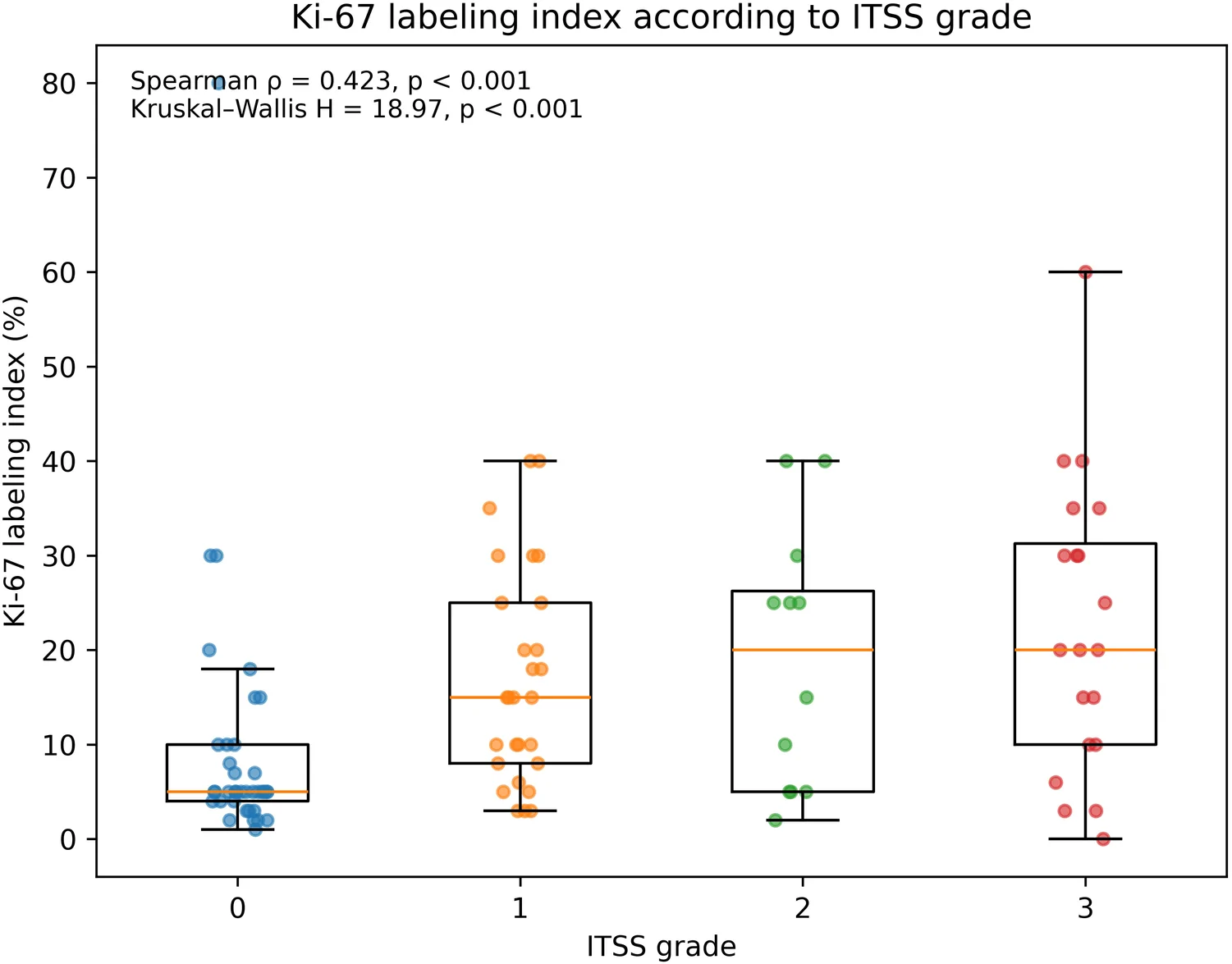
Median ki-67 LI significantly increases with higher ITSS grades, demonstrating that elevated ITSS correlates with higher tumor proliferation activity. Notably, ITSS grades 2 and 3 show greater variability and higher median proliferation indices compared to grades 0 and 1.

### 3.5 ITSS and survival data

For survival analysis, patients with oligodendroglioma, IDH-mutant and 1p/19q-codeleted (*n* = 19), were excluded because no death events occurred during follow-up. Survival analyses were restricted to patients with IDH-mutant astrocytomas and IDH-wildtype gliomas, among these 22 patients were excluded because follow-up information was insufficient to reliably determine survival time or censoring. The final OS cohort comprised 87 patients, including 42 deaths and 45 censored observations. In the overall OS cohort, higher ITSS burden was significantly associated with shorter OS (Figure 4). Survival differed significantly across the four individual ITSS grades (log-rank *p* = 0.003). When ITSS was dichotomized into low (grades 0–1) and high (grades 2–3) categories, patients with high ITSS burden showed significantly shorter OS than those with low ITSS burden (log-rank *p* < 0.001).

**Figure 4:**
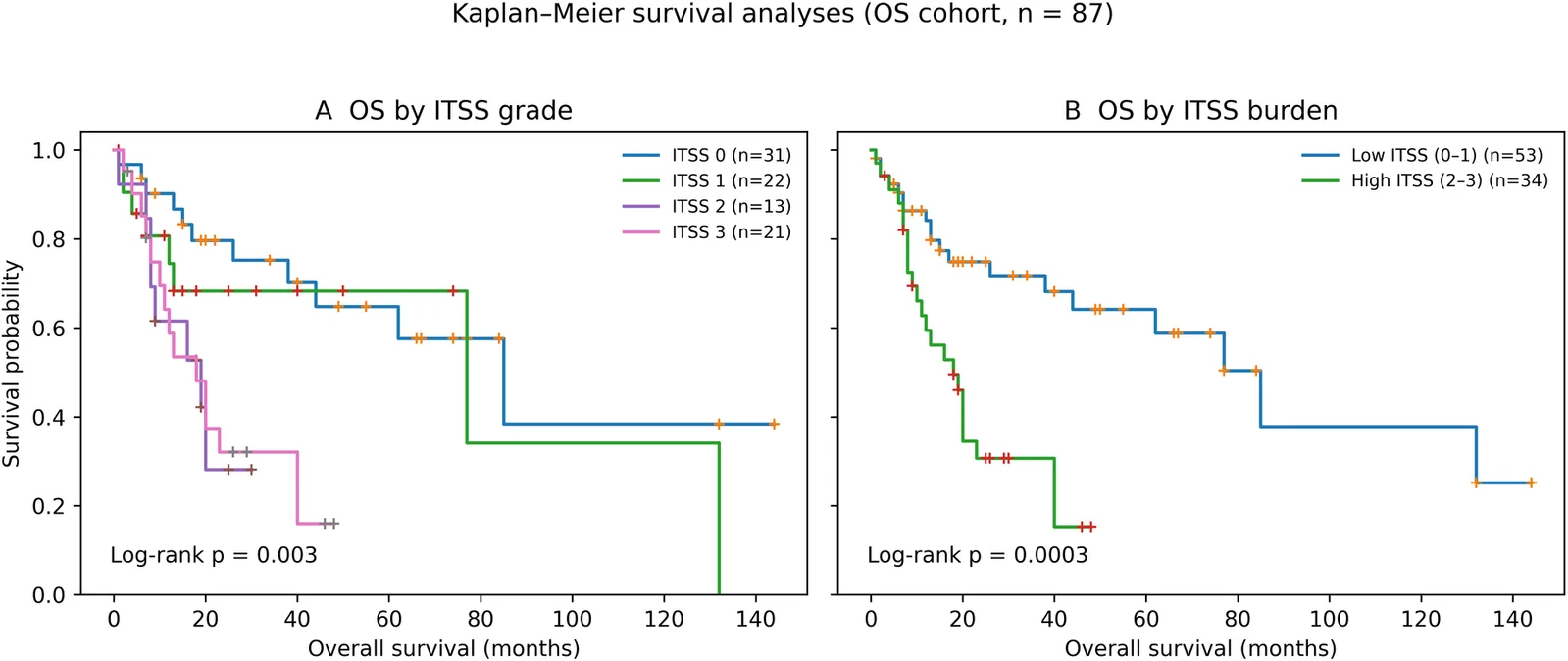
Kaplan–meier analysis of overall survival according to ITSS grade in adult diffuse gliomas. Survival analysis was performed in the final OS cohort of 87 patients (42 deaths and 45 censored observations). **(A)** Overall survival (OS) according to the four individual ITSS grades (0–3), showing significant differences in survival across ITSS categories (log-rank *p* = 0.003). **(B)** OS after dichotomization into low (ITSS grades 0–1) and high (ITSS grades 2–3) ITSS groups, showing significantly shorter survival in patients with high ITSS grades (log-rank *p* < 0.001). Censoring is indicated by tick marks.

However, this association was not maintained after stratification by established prognostic categories (Supplementary Figure S1). Within IDH-mutant astrocytomas and IDH-wildtype gliomas, OS did not differ significantly between low and high ITSS groups (log-rank *p* = 0.164 and *p* = 0.393, respectively). Similarly, ITSS did not significantly stratify OS within individual CNS WHO grade groups (grade 2, *p* = 0.432; grade 3, *p* = 0.206; grade 4, *p* = 0.729).

In univariate Cox proportional hazards analysis (Table 4), high ITSS grade (grades 2–3 vs. 0–1) was significantly associated with shorter OS (HR = 3.19, 95% CI: 1.65–6.19; *p* < 0.001). ITSS was also significantly associated with OS when analyzed as an ordinal variable, with a 57% increase in the hazard of death for each one-grade increase in ITSS (HR = 1.57, 95% CI: 1.22–2.04; *p* < 0.001).

**Table 4:** Cox proportional hazards regression analyses for overall survival.

Model/Variable	HR	95% CI	*p*-value
Univariate analyses			
ITSS high vs low (2–3 vs 0–1)	3.19	1.65–6.19	<0.001
ITSS, per 1-grade increase	1.57	1.22–2.04	<0.001
Multivariable Model 1			
ITSS 2–3 vs 0–1	1.40	0.69–2.86	0.350
IDH-wildtype	5.99	2.10–17.11	<0.001
Age, per year	1.027	0.999–1.057	0.063
Male sex	0.67	0.34–1.32	0.248
Multivariable Model 2			
ITSS 2–3 vs 0–1	1.33	0.59–2.97	0.491
CNS WHO grade, per grade increase	1.67	0.96–2.89	0.069
Age, per year	1.039	1.012–1.067	0.004
Male sex	0.88	0.46–1.71	0.710

ITSS was significantly associated with OS in univariate analyses, both when dichotomized into high (grades 2–3) versus low (grades 0–1) burden and when modeled as an ordinal variable. In multivariable analyses, the association between ITSS and OS was attenuated after adjustment for established prognostic factors. IDH-wildtype status was independently associated with poorer OS in Model 1, whereas older age was independently associated with poorer OS in Model 2. HR, hazard ratio; CI, confidence interval; ITSS, intratumoral susceptibility signals; IDH, isocitrate dehydrogenase.

In the multivariable model including ITSS category, IDH mutation status, age, and sex, ITSS was no longer significantly associated with OS (HR = 1.40, 95% CI: 0.69–2.86; *p* = 0.350), whereas IDH-wildtype status remained independently associated with shorter OS (HR = 5.99, 95% CI: 2.10–17.11; *p* < 0.001). Age (HR = 1.027 per year, 95% CI: 0.999–1.057; *p* = 0.063) and sex (HR = 0.67, 95% CI: 0.34–1.32; *p* = 0.248) were not significantly associated with OS in this model. In the separate model including ITSS category, CNS WHO grade, age, and sex, ITSS was again not independently associated with OS (HR = 1.33, 95% CI: 0.59–2.97; *p* = 0.491). Increasing CNS WHO grade showed a borderline association with shorter OS (HR = 1.67, 95% CI: 0.96–2.89; *p* = 0.069), whereas older age was independently associated with shorter OS (HR = 1.039 per year, 95% CI: 1.012–1.067; *p* = 0.004). Sex was not significantly associated with OS (HR = 0.88, 95% CI: 0.46–1.71; *p* = 0.710).

## 4 Discussion

ITSS, presence and extent, have been associated with microvascular density and hemorrhagic components, including abnormal, tortuous vessels and microhemorrhages, and have therefore been investigated as imaging markers of glioma aggressiveness (20, 21). Previous studies have shown that ITSS may contribute to the differentiation of high-grade gliomas (HGGs), from other neoplastic and non-neoplastic lesions, with higher ITSS burden generally observed in more aggressive tumors (22–25).

In our cohort, ITSS grade differed significantly across molecular glioma subtypes, with higher ITSS grades more frequently observed in glioblastoma, *IDH*-wildtype, whereas lower ITSS grades were more typical of astrocytoma, *IDH*-mutant. These findings are consistent with previous susceptibility-based imaging studies reporting higher ITSS grade in *IDH*-wildtype tumors (26–29). Our data demonstrate an association between ITSS grade and *IDH* status. Previous studies have proposed that the greater susceptibility burden observed in *IDH*-wildtype gliomas may be related to features of their more aggressive phenotype, including abnormal tumor vascularity, necrosis, hypoxia, and microhemorrhage (26–29). Oligodendrogliomas (*IDH*-mutant and 1p/19q-codeleted) showed a different ITSS distribution, with grade 1 being particularly frequent. Interpretation of this finding requires caution because of the relatively small number of oligodendrogliomas in our cohort. Calcification should also be considered when interpreting susceptibility-related intratumoral hypointensities in this subtype, given its relatively high prevalence in oligodendrogliomas (30–32). In the present study, however, susceptibility imaging was performed using the GE SWAN (T2 Star Weighted ANgiography) multi-echo technique, which provides both magnitude and phase information. Phase information allows diamagnetic susceptibility sources such as calcium to be differentiated from paramagnetic blood products and iron, thereby reducing the potential confounding effect of calcification, including at 1.5 T. Nevertheless, systematic CT correlation or quantitative susceptibility mapping (QSM) could provide a more quantitative characterization of susceptibility sources in future studies. In contrast, *IDH*-mut astrocytomas more commonly exhibited grade 0 ITSS, consistent with their lower proliferative and vascular profiles.

No statistically significant association was observed between ITSS grade and MGMT promoter methylation status (*p* = 0.065). Although lower ITSS grades tended to be more frequent in MGMT-methylated tumors and higher grades in unmethylated tumors, these findings should be interpreted as descriptive trends rather than evidence of a specific association. This differs from susceptibility-based observations reported in some previous studies (29, 33) and suggests that ITSS cannot reliably serve as an imaging surrogate for MGMT status. More broadly, ITSS should not be considered a substitute for molecular characterization, but rather as a complementary imaging feature that may contribute to preoperative tumor characterization. Its interpretation within a multiparametric MRI framework, together with diffusion, perfusion, spectroscopy, and established imaging markers such as the T2-FLAIR mismatch sign, may provide more comprehensive information regarding tumor phenotype (34).

A significant association was also observed between ITSS grade and CNS WHO grade, in agreement with previous studies (27, 35). Lower ITSS grades predominated among lower-grade tumors, whereas ITSS grade 3 was mainly observed in CNS WHO grade 4 gliomas, supporting the premise that high-grade tumors exhibit greater intralesional vascular disruption and microhemorrhages detectable on SWI. When tumors were stratified by histological subtype, this association remained significant in astrocytomas but not in oligodendrogliomas. The latter finding should be interpreted cautiously given the limited size of the oligodendroglioma subgroup and its different susceptibility characteristics, including the potential contribution of calcification (31). Nevertheless, the absence of ITSS grade 0 in grade 2 oligodendrogliomas, and the confinement of ITSS values between grades 0–2 in grade 3 tumors, could suggests a more homogeneous pattern compared with astrocytomas.

A strong association was observed between ITSS grade and contrast enhancement, with enhancement occurring more frequently in tumors with higher ITSS grades. However, the two imaging features were not interchangeable. Notably, ITSS were also detected in 8 of 41 (19.5%) non-enhancing tumors, including five tumors with ITSS grade 1 and three with ITSS grade 2. Moreover, our analysis was performed at the lesion level and did not assess spatial co-localization; particularly in high-grade gliomas with peripheral or ring-like enhancement, susceptibility signals may also be observed within non-enhancing or necrotic tumor components. These findings indicate that ITSS and contrast enhancement represent related but distinct imaging features. While contrast enhancement reflects blood–brain barrier disruption and has traditionally been associated with more aggressive tumor behavior and poorer outcome (36), susceptibility imaging may capture additional aspects of tumor microstructure that are not necessarily accompanied by contrast enhancement.

Our study also demonstrated a significant positive association between ITSS grade and Ki-67 labeling index. Ki-67 values increased across ITSS categories, and this relationship was confirmed using Spearman's rank correlation (*ρ* = 0.423, *p* < 0.001) and the Kruskal–Wallis test (H = 18.97, *p* < 0.001). These results indicate that higher ITSS burden is associated with greater proliferative activity. Although the biological mechanisms responsible for this association were not directly investigated in the present study, previous literature suggests a plausible link between tumor proliferation, hypoxia, angiogenic activation, and susceptibility-related imaging findings (29, 37). Hypoxia-driven pathways involving HIF-1*α* and VEGF may promote abnormal and fragile tumor vasculature, potentially resulting in microhemorrhage and increased deoxygenated blood detectable on susceptibility-sensitive imaging (38, 39).

To the best of our knowledge, this study represents one of the largest molecularly characterized cohorts evaluating the association between ITSS and survival outcomes in adult diffuse gliomas. Because no deaths occurred in the oligodendroglioma subgroup, survival analyses were restricted to patients with *IDH*-mutant astrocytomas and *IDH*-wildtype gliomas. In this cohort, increasing ITSS grade was significantly associated with shorter OS. Patients with high ITSS burden (grades 2–3) had a significantly higher risk of death than those with low ITSS grade (grades 0–1) in univariate Cox analysis (HR = 2.66, 95% CI: 1.42–4.97; *p* = 0.002). Similarly, when ITSS was analyzed as an ordinal variable, each one-grade increase was associated with a 47% increase in the hazard of death (HR = 1.47, 95% CI: 1.16–1.88; *p* = 0.002). Subgroup analyses, however, provided a more nuanced interpretation of this association. ITSS did not significantly stratify survival within IDH-mutant astrocytomas or IDH-wildtype gliomas, nor within individual WHO grade groups. Consistently, the association between ITSS and OS was attenuated and no longer statistically significant in multivariable models adjusted for either IDH mutation status or WHO grade together with age and sex. IDH-wildtype status emerged as the strongest independent predictor of poorer outcome in the molecular model. Taken together, these findings suggest that the association between ITSS and survival is closely related to the underlying molecular and histopathological aggressiveness of the tumor rather than reflecting a fully independent prognostic effect. This interpretation is biologically plausible, as a higher ITSS burden may reflect features associated with aggressive glioma biology, including abnormal angiogenesis, hypoxia, microvascular proliferation, and intratumoral hemorrhage.

Only a limited number of studies have specifically investigated susceptibility-based imaging markers in relation to survival in diffuse gliomas (29, 40). Shen et al. (29), using QSM, reported an association between susceptibility burden and OS, with lower susceptibility values associated with more favorable outcome. Our results are broadly consistent with the observation that susceptibility burden is related to prognosis, but provide an important qualification: in our cohort, ITSS was associated with OS in univariate analysis but not after adjustment for major prognostic factors. Thus, ITSS appears more appropriately regarded as an imaging correlate of an aggressive tumor phenotype than as an independent prognostic biomarker. The present study also differs methodologically from QSM-based approaches. QSM provides quantitative characterization of tissue magnetic susceptibility but requires dedicated acquisition and post-processing and is not universally incorporated into routine neuro-oncological MRI protocols. In contrast, semiquantitative ITSS grading can be performed directly on routinely acquired susceptibility-sensitive images. This accessibility may represent its principal clinical advantage. ITSS could therefore provide an early, non-invasive estimate of tumor aggressiveness during the preoperative diagnostic work-up, when definitive histopathological and molecular information is not yet available. Its potential value should nevertheless be considered complementary to, rather than a replacement for, established molecular and histopathological markers.

From a clinical perspective, susceptibility-sensitive imaging may also provide information relevant to procedural planning. Previous studies have suggested potential applications of ITSS in biopsy targeting and hemorrhagic risk assessment, with high ITSS burden reported as a predictor of significant hemorrhage during stereotactic brain biopsy (41–43). These applications were not evaluated in the present study and therefore represent potential clinical implications derived from previous literature rather than conclusions supported directly by our data. Future multicenter studies with standardized susceptibility imaging protocols, larger molecularly defined cohorts, and integration with quantitative and radiomic approaches will be required to determine the incremental clinical value of ITSS within contemporary neuro-oncology workflows.

### 4.1 Limitations and future directions

Several limitations should be acknowledged. First, the retrospective single-center design may have introduced selection bias. In particular, a proportion of otherwise eligible patients had to be excluded because SWI was not available in the preoperative MRI examination, potentially affecting cohort composition. Second, the number of oligodendrogliomas was relatively limited, reducing the statistical power of subgroup analyses and potentially contributing to the lack of significant associations between ITSS and tumor grade in this molecular subtype. Molecular characterization was performed according to the standards available at the time of diagnosis, and information regarding CDKN2A/B homozygous deletion was not systematically available. Consequently, a proportion of tumors currently classified according to the WHO CNS5 criteria may not have undergone complete molecular profiling by contemporary standards. Future prospective studies incorporating comprehensive contemporary molecular characterization will be important to further define the relationship between ITSS and glioma phenotype. The semiquantitative ITSS grading system (15) is inherently limited by its single-slice assessment and some degree of reader dependence. Nevertheless, the high weighted inter-rater agreement observed in our study (*κ* = 0.85) supports the reproducibility of this approach. Volumetric ITSS assessment and automated quantification techniques (20, 44) may further improve objectivity and should be evaluated in future studies. Although phase information from the SWAN acquisition reduced the potential confounding effect of calcification, more advanced susceptibility-based techniques such as QSM may provide more detailed and quantitative characterization of tissue magnetic susceptibility (45, 46). However, these methods require dedicated post-processing and additional computational resources and remain less readily available than conventional semiquantitative ITSS assessment in routine clinical practice. Lastly survival analyses were not adjusted for treatment-related variables, including extent of resection, adjuvant radiotherapy, chemotherapy, or treatment at recurrence. Given the retrospective nature of the study and the long inclusion period, therapeutic approaches were heterogeneous. Therefore, the observed association between ITSS and OS should be interpreted as reflecting the relationship between ITSS and tumor biological aggressiveness rather than as evidence of an independent treatment-adjusted prognostic effect.

## 5 Conclusions

Semiquantitative assessment of ITSS on susceptibility-sensitive MRI provides a non-invasive imaging marker associated with relevant histopathological and molecular features of adult-type diffuse gliomas. In our cohort, increasing ITSS burden was significantly associated with higher CNS WHO grade, *IDH*-wildtype status, and higher Ki-67 labeling index. Higher ITSS burden was also associated with shorter OS in univariate analyses; however, this association was attenuated after adjustment for established prognostic factors and was not significant within individual *IDH*- or CNS WHO grade-defined subgroups. The absence of independent prognostic significance does not preclude the potential clinical value of ITSS as a preoperative imaging biomarker. Rather, ITSS may provide a non-invasive imaging representation of an aggressive tumor phenotype associated with poorer outcome. Unlike histopathological and molecular prognostic markers, which become available only after tissue sampling, ITSS can be assessed on SWI during the initial diagnostic work-up. Thus, ITSS may contribute to an early imaging-based estimate of tumor aggressiveness and associated prognosis, complementing other MRI findings while definitive molecular characterization is still pending.

ITSS can be readily assessed on susceptibility-sensitive sequences routinely incorporated into MRI protocols for brain gliomas. Although susceptibility effects are more conspicuous at higher magnetic field strengths, the present findings demonstrate that semiquantitative ITSS assessment is feasible and reproducible at 1.5 T, supporting its potential applicability in routine clinical settings. Future multicenter prospective studies with larger molecularly defined cohorts, standardized susceptibility imaging protocols, and quantitative or automated approaches are needed to validate these findings and determine the incremental clinical and prognostic value of ITSS within contemporary neuro-oncology imaging. While challenges remain, particularly regarding technical limitations and the lack of a standardized model for quantifying ITSS, advancements in imaging technology, computational analysis, and longitudinal studies with larger cohorts are expected to further define its clinical utility for pre- and post-treatment evaluation of brain gliomas.

## Data Availability

The raw data supporting the conclusions of this article will be made available by the authors, without undue reservation.

## Supplementary material

Supplementary Figure S1 Kaplan-Meier analysis of overall survival according to ITSS category within molecular and CNS WHO grade-defined subgroups. Overall survival (OS) was compared between low (ITSS grades 0–1) and high (ITSS grades 2–3) ITSS groups within the final OS cohort. (A) IDH-mutant astrocytomas (n=38; log-rank p=0.164); (B) IDH-wildtype gliomas (n=49; p=0.393); (C) CNS WHO grade 2 gliomas (n=22; p=0.432); (D) CNS WHO grade 3 gliomas (n=24; p=0.206); and (E) CNS WHO grade 4 gliomas (n=41; p=0.729). No statistically significant differences in OS between low and high ITSS groups were observed within any of the molecular or CNS WHO grade-defined subgroups. Censoring is indicated by tick marks.
